# Supplementary material for: Content-rich biological network constructed by mining PubMed abstracts
Source: BMC Bioinformatics. 2004 Oct 8;5:147. doi: 10.1186/1471-2105-5-147 (PMC528731; doi:10.1186/1471-2105-5-147)
Supplement: Additional File 2 — The original results of the above study (non-essential files are deleted to keep the file size under the limit set by BMC bioinformatics). [file 1471-2105-5-147-S2.bz2 › chilibotAdditionalFile2/dip05/502ID7878020E10668/html/TGFB3_TGFBR2.html]

 


 **TGFB3** and **TGFBR2** 
  
Found 31 abstracts in PubMed, retrieved 05.  
 

 What does Google say? 
 PDF only 
| .edu only 

---

**Interactive relationship** (e.g. stimulation, inhibition, etc)

**Non-interactive relationship** (e.g. studied together, co-existance, homology, etc.)

- Here, we present the 2.15 A crystal structure of the extracellular ligand binding domain of the human TGF beta type II receptor  [ **TGFBR2** ]  ecTbetaR2 in complex with human TGF beta3  [ **TGFB3** ] .  Ref: 11850637 Nat Struct Biol, 2002
- We therefore studied the expression of UPA, UPAR, IGF2R, ALK5,  **TGFBR2** , TGFBR3, ENG, ALK1, TGFB1, TGFB2, and  **TGFB3**  in a series of 14 pancreatic carcinoma cell lines.  Ref: 11408925 Int J Oncol, 2001
